# Association between Visit-to-Visit Ultrafiltration Volume Variability, Vascular Biomarkers and Cardiovascular Parameters in Chronic Hemodialysis Patients

**DOI:** 10.3390/jcm13195958

**Published:** 2024-10-07

**Authors:** Balázs Sági, Tibor Vas, Rita Klaudia Jakabfi-Csepregi, Endre Sulyok, Botond Csiky

**Affiliations:** 1 2nd Department of Internal Medicine and Nephrology, Diabetes Center, Clinical Center, Medical School, University of Pécs, Pacsirta Street 1, 7624 Pécs, Hungary; balazs.sagidr28@gmail.com (B.S.); vas.tibor@pte.hu (T.V.); 2 National Dialysis Center Pécs, 7624 Pécs, Hungary; 3 Institute of Laboratory Medicine, Medical School, University of Pécs, 7624 Pécs, Hungary; ritacsepregi93@gmail.com; 4 Szentágothai Research Center, Molecular Medicine Research Group, University of Pécs, 7624 Pécs, Hungary; 5 Doctoral School of Health Sciences, University of Pécs, 7624 Pécs, Hungary; esulyok@t-online.hu

**Keywords:** hemodialysis, syndecan-1, endothelin-1, ultrafiltration volume variability, arterial stiffness, LVMI

## Abstract

**Background.** Cardiovascular (CV) diseases are the most common causes of morbidity and mortality in hemodialysis (HD) patients. We studied the effect of high visit-to-visit ultrafiltration (UF) variability on CV abnormalities in HD patients. **Methods.** Twenty-nine consecutive patients (age: 65.6 ± 10.4 years) were recruited. Samples for routine lab tests were drawn pre-HD for syndecan-1 (SDC-1) and endothelin-1 (ET-1) measurements pre-, mid- and post-HD. Applanation tonometry was performed pre-, mid- and post-HD. Visit-to-visit ultrafiltration volume variability (UVSD) was calculated as the standard deviation of the UF volume/dialysis session in the preceding 12 months. Echocardiography was performed post-HD. **Results.** Patients were divided into two groups based on the median of UVSD (500 mL). The average UF volume/HD was not different between the groups. Blood pressure (BP) values were similar. Pre-HD cfPWV (10.75 m/s) was lower in the high UVSD group (14.1 m/s, *p* = 0.03). In the high UVSD group, post-HD cfPWV (13.9 m/s) was higher than the pre-HD cfPWV (*p* < 0.05). Pre-HD ET-1 was lower in the high UVSD group (8.6 ± 3.9 vs. 10.8 ± 2.7 pg/mL, *p* < 0.05). Left ventricular end-diastolic diameter (LVEDD) and left ventricular mass index (LVMI) were higher in the high UVSD group (55.7 ± 7.3 vs. 51.0 ± 5.4 mm and 449.9 ± 180.5 vs. 350.3 ± 85.9 g/m², *p* < 0.005, respectively). Left ventricular ejection fraction (LVEF) was lower in the high UVSD group (53.5 vs. 60, *p* < 0.05). **Conclusions.** High UVSD was associated with increased left ventricular hypertrophy and dysfunction and decreased LVEF compared to low visit-to-visit UV variability despite similar UF volumes temporarily compensated by more elastic arteries. The observed abnormalities may increase CV risk.

## 1. Introduction

The global incidence and prevalence of chronic kidney disease (CKD) is on the rise, with an estimated 160 million patients worldwide [1,2,3]. It is crucial to recognize that cardiovascular (CV) disease is the leading cause of morbidity and mortality in CKD patients. Both traditional risk factors (hypertension, diabetes, dyslipidemia, obesity and smoking) and non-traditional risk factors (volume overload, anemia, calcium phosphate metabolism disorders, hyperkalemia and chronic inflammation) play a significant role [4,5,6]. Notably, patients with end-stage kidney disease (ESKD) face a considerably higher risk of CV death compared to the general population [7,8]. Oxidative stress contributes to the development of vascular dysfunction and coronary artery disease [9] and increased arterial stiffness [10], further exacerbated by the bone–vascular interaction [11,12]. While the salt- and volume-dependent hypertension of chronic hemodialysis (HD) patients and the hypotension resulting from ultrafiltration (UF) have been extensively studied, their pathophysiology remains incompletely understood. Pressure overload (caused by hypertension or decreased vascular compliance) is a major contributor to the development of left ventricular hypertrophy (LVH) in ESKD. Increased preload (caused by renal anemia, salt- and volume overload and/or high-output arterial-venous dialysis access) is similarly important in the development of LVH in HD patients [13]. High interdialytic weight gain demonstrating considerable hypervolemia and necessitating high UF volume during HD sessions also contributes to the hypertrophy of the left ventricle. The majority of studies have demonstrated strong associations between LVH/left ventricular mass index (LVMI) and survival/CV outcomes. The presence of LVH is a strong risk factor for adverse outcomes in HD patients [14,15]. On the other hand, high UF volume or high UF rate during HD is also associated with intradialytic hypotension, myocardial stunning and higher CV morbidity and mortality. An association between reduced left ventricular ejection fraction (LVEF) and increased adverse events in HD patients has been described. It has been proven that both hypertension and post-dialysis hypotension have a negative effect on survival in these patients [16].

Cardiac and vascular abnormalities are developing in close interrelationships in HD patients. Endothelial factors are important in controlling the endothelial tone and, by that, the blood pressure.

Endothelial glycoprotein (GCX) is a vital protective barrier lining the inner side of the endothelium, shielding it against pathogenic damage. It plays a crucial role in maintaining vascular permeability, preventing inflammation and inhibiting the development of atherosclerosis. Additionally, it is instrumental in the synthesis of nitric oxide (NO). However, its integrity can be compromised by end-stage kidney disease (ESKD) [17,18]. Plasma syndecan-1 (SDC-1) is a key marker of endothelial GCX degradation, and elevated levels are indicative of endothelial injury. SDC-1 not only safeguards the endothelial GCX from harm but also facilitates the attachment of platelets, leukocytes and inflammatory cells to the endothelial surface. It holds great promise as a biomarker for the diagnosis and prognosis of vascular diseases [19,20]. It is a potent vasoconstrictor. It might also additionally play a position in CV illnesses, which include hypertension, myocardial infarction and stroke. ET-1 plays an important role in cell migration, cell division, regulation of cell division, regulation of cell functions and maintenance of cell composition [21,22,23].

The detrimental CV effect of considerable interdialytic overhydration and high UF during dialysis sessions is well known. Little is known about the effect of visit-to-visit ultrafiltration volume variability (UVSD) on the cardiac and arterial complications developing in chronic HD patients, although in one study, it has been described that it may predict all-cause mortality in this patient group [24].

We hypothesized that high UVSD may have adverse CV effects mediated in part by endothelial factors like SDC-1 and ET-1.

Our study aimed to describe the effect of UVSD on central and peripheral blood pressure (BP), augmentation index (Aix), carotid-femoral pulse wave velocity (cfPWV) and cardiac abnormalities studied by echocardiography.

## 2. Materials and Methods

### 2.1. Patients

In our comprehensive study at the TritonLife Dialysis Center of Pécs from January 2022 to March 2023, we included 29 consecutive adult chronic HD patients who had been part of the dialysis program for at least 3 months. Excluded from the study were patients with lower extremity amputations, any acute or chronic infection, malignancy, acute myocardial infarction, pulmonary edema or hemodynamic instability.

The underlying renal pathologies that led to ESKD were diabetic nephropathy (8), nephrosclerosis (14), chronic glomerulonephritis (3), polycystic kidney disease (2), chronic interstitial nephritis (1) and other/unknown causes (1).

A majority of the patients (27) received antihypertensive therapy. The patients underwent 3 HD sessions per week, each lasting for 4 h. Online hemodiafiltration was carried out using Fresenius 5008 B equipment with Helixone/Fresenius polysulfone high-flux dialyzer membranes. The body mass index (BMI) was calculated.

Blood pressure, pulse wave velocity, echocardiography and multifrequency bioimpedance measurements were measured. 

Blood pressure was measured with the Fresenius 5008S integrated blood pressure module, which is a calibrated automated device that comes with appropriate cuff sizes. Applanation tonometry (SphygmoCor system, AtCor Medical Australia) was used to measure carotid-femoral pulse wave velocity (cfPWV), augmentation index (AIx) and central/aortic BP. Pre-, mid- and post-dialysis measurements were taken. Every recorded value met the quality control requirements of the manufacturer’s software. Following the HD session, a routine echocardiography examination was performed.

The patient’s fluid status was determined before the dialysis with multifrequency bioimpedance measurement (Body Composition Monitor, Fresenius Medical Care^®^). Overhydration was calculated with the manufacturer’s software based on the measured parameters.

Visit-to-visit ultrafiltration volume variability (UVSD) was calculated as the standard deviation of the ultrafiltration volume/dialysis session in the 12 months preceding the HD treatment when the above measurements were performed. The average number of HD sessions in 12 months was 150 (119–158).

### 2.2. Laboratory Measurements

Routine biochemical parameters were assessed using standard methods prior to dialysis. Serum ET-1 and SDC-1 were analyzed using human enzyme-linked immunoassay (ELISA) kits from Sigma-Aldrich Chemie GmbH, Taufkirchen, Germany, in samples obtained before, during and after HD. Blood was collected through venipuncture in ethylene-diamine-tetra-acetic acid (EDTA) tubes pre-, mid- and post-HD. Plasma was obtained by centrifuging blood at 1300× *g* for 10 min at 4 °C and then stored at −70 °C. Concentrations of soluble syndecan-1 were determined by a commercially available human syndecan-1enzyme-linked immunoassay (ELISA) kit (Sigma-Aldrich Chemie GmbH, Taufkirchen, Germany). The sensitivity of the assay was <2.56 ng/mL, and the coefficients of intra-assay variation and inter-assay variation were 7.6% and 6.8%, respectively.

Plasma ET-1 levels were measured at Szentágothai Research Center, Molecular Medicine Research Group, University of Pécs, using a commercially available ELISA kit (Sigma-Aldrich Chemie GmbH, Taufkirchen, Germany). The mean inter-assay coefficient of variation was 7%.

### 2.3. Echocardiography

All patients underwent a clinically indicated 2D echocardiogram. Echocardiography was performed with Aloka SSD 1400, and standard echocardiographic images in the parasternal, apical and subcostal views in the left lateral decubitus position were obtained by two experienced cardiologists. Left ventricular mass (LVM) was calculated from 2D images of the left ventricular short-axis muscle area and apical left ventricular length (LVM = (5/6 area × length)). The assessment of left ventricular mass index (LVMI g/m^2^) was obtained according to the formula of Devereux; the cardiac mass was also indicized with lean mass. LVMI was determined based on the Cornell criterion and indexed for height (in meters). The left ventricular ejection fraction (LVEF) was calculated by calculating the diastolic and systolic left ventricular volumes using the unidirectional Simpson method: EF = ((Dvol-Svol)/Dvol) × 100. Measurement of left atrial volume (LAV) from the prolate ellipse method was obtained using the apical 4-chamber and parasternal long-axis views at ventricular end-systole (maximum LA size). Right atrial volume (RAV) was measured using Simpson’s method in the apical four-chamber view.

### 2.4. Statistical Analysis

The results are displayed along with a frequency and an average range of confidence. We assessed the normality of the data by conducting the Kolmogorov–Smirnov test. Data were presented as mean ± standard deviation (SD) in cases of normal distribution and as median (lower or upper quartile) in cases of non-normal distribution. Between-group comparison was performed using a *t*-test in normally distributed continuous parameters, while the Mann–Whitney U test was used to compare parameters with non-normal distribution. We used the Pearson test to determine correlations between continuous variables through linear regression. The significance level was determined at *p* < 0.05. Statistical analyses were performed using SPSS (SPSS, Inc., Chicago, IL, USA) version 21.0 software.

### 2.5. Ethical Consideration

The Regional Ethics Committee of the Medical School of Pécs approved the research. The study complies with the guidelines provided in the Declaration of Helsinki of the World Medical Association. Every individual involved provided written informed consent.

## 3. Results

The baseline clinical data of the 29 chronic HD patients are summarized in Table 1.

Patients were divided into two groups based on the median value of the visit-to-visit UVSD (UVSD < 500 mL vs. UVSD ≥ 500 mL). The two groups had mostly similar parameters. CRP was significantly higher in the high UVSD group, and dialysis vintage was significantly higher in the low UVSD group. Despite the difference in the UVSD, the average UF volume/HD session was not different between the two groups. Similarly, the average UF volume/body weight and the overhydration assessed by multifrequency bioimpedance measurement were also similar in the two groups (Table 1).

We found no difference in the brachial BPs between the groups. Mid-HD central/aortic systolic BP was significantly lower in the high UVSD group. The difference between the pre-HD and mid-HD aortic systolic BP in this group was not significant.

There was no significant difference in Aix between the groups. On the other hand, pre-HD cfPWV was significantly lower in the high UVSD group (10.75 m/s) than in the low UVSD group (14.1 m/s). In the high UVSD group, post-HD cfPWV (13.9 m/s) was higher than the pre-HD cfPWV (10.75 m/s) (*p* < 0.05).

We found no significant difference in SDC-1 levels between the groups. Pre-HD ET-1 was significantly lower in the high UVSD group. Mid- and post-HD ET-1 levels were not different.

Regarding the echocardiographic parameters, LVEDD and LVMI were significantly higher in the high UVSD group. LVEF was lower in the high UVSD group than in the low UVSD group (Table 2).

**Figure 1:**
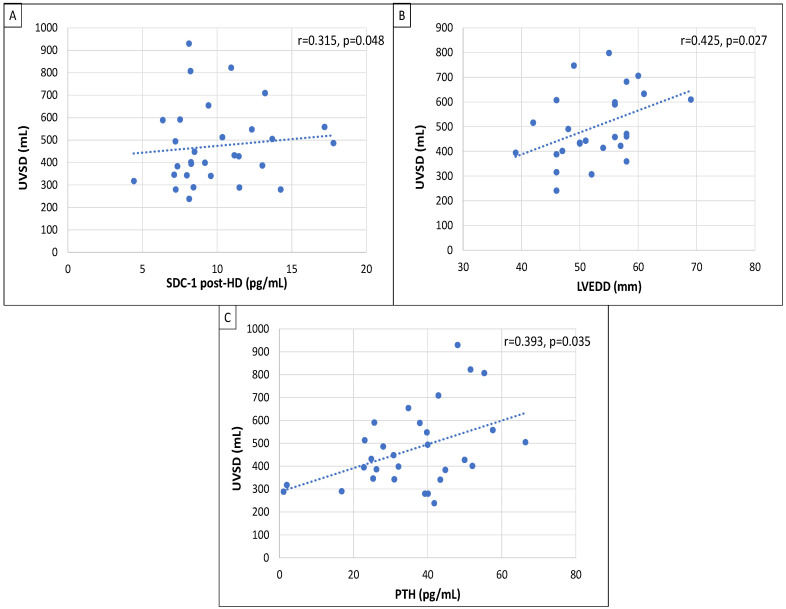
Pearson’s correlations between UVSD and SDC-1 post-HD (**A**); LVEDD (**B**); PTH (**C**). UVSD: ultrafiltration volume standard deviation; SDC-1: syndecan-1; LVEDD: left ventricular end-diastolic diameter; PTH: parathormone.

Correlation analysis was performed with the values of UVSD in the original patient group (*n* = 29). UVSD had strong positive correlation with post-HD SDC-1 (r = 0.315; *p* = 0.048), LVEDD (r = 0.425; *p* = 0.027) and PTH (r = 0.393; *p* = 0.035; Figure 1).

## 4. Discussion

HD patients have increased CV morbidity and mortality. Volume overload is highly prevalent among these patients. Excessive interdialytic weight gain and the consequent excessive intradialytic weight loss constitute a cyclical stress on the CV system, contributing to the high CV risk in these patients [25]. It has been known that high UF volume has a detrimental effect on the CV system [26] in HD patients. In this study, we investigated the effect of high visit-to-visit UF variability on the heart and the arteries. We compared two patient groups having similar average UF volume measured and also assessed by multifrequency bioimpedance measurement but different visit-to-visit UF variability in the 12 months preceding our examinations. We hypothesized that high visit-to-visit UF variability may have adverse CV effects irrespective of the UF volume.

We found that despite similar peripheral BP values, pre-HD cfPWV was lower in the high UVSD than in the low UVSD group. The cfPWV remained unchanged during dialysis in the low UVSD group and increased significantly from 10.75 (pre-HD) to 13.9 m/s (post-HD) in the high UVSD group. According to our results, patients in the latter group seem to have more elastic arteries capable of accommodating the frequent changes of the intravascular volume. The cfPWV is known to be influenced by the BP [27], but pre-HD peripheral and central BP values were similar in the compared groups, so we assume that the observed difference in the pre-HD cfPWV demonstrates different arterial stiffness. Dialysis vintage in the high UVSD patients was significantly less than in the other group. It has been demonstrated that the stiffness of the arteries increases with the time spent on HD [28], so in this regard, our findings are congruent with the literature.

We assumed that higher UVSD causes higher shear stress and endothelial injury on the arterial wall than lower UVSD, and we hypothesized that the endothelial injury would be detectable by higher SDC-1 levels in the high UVSD group. According to the literature, SDC-1 levels are increasing when endothelial damage occurs [23,29]. On the other hand, SDC-1 has also been described as a marker of endothelial repair. We could not demonstrate any difference in the SDC-1 levels between the examined patient groups, but we have found a strong positive correlation between UVSD and post-HD SDC-1, demonstrating that the high fluctuation of the intravascular volume may cause endothelial injury and/or may necessitate intensive endothelial repair.

ET-1 is a potent endothelium-derived vasoconstrictor peptide implicated in the pathogenesis of hypertension and inflammation [30,31]. ET-1 in HD patients has been described to correlate with the patient’s BP, volume status and UF volume [32]. In our patient groups, average peripheral and central BP, volume status (overhydration measured by bioimpedance analysis) and the average UF volume were similar. On the other hand, the average pre-HD ET-1 level was lower in the high UVSD group. The average ET-1 levels did not change during the HD session in our patients, fitting the literature stating that ET-1 does not change during HD in normally hydrated patients who do not have intradialytic hypotension or hypertension [33,34]. Increased production of ET-1 may promote oxidative stress, development of endothelial dysfunction and increased arterial stiffness [35]. Lower pre-HD ET-1 in our high UVSD group can be interpreted as a sign of less arterial stiffening compared to the low UVSD group, in line with the lower cfPWV in this group, demonstrating again that the arteries are more elastic in high UVSD patients.

PTH is a well-known CKD-specific non-traditional CV risk factor. High PTH has an important role in the calcification of the medial layer of the arteries, causing decreased distensibility [36]. We have found a significant positive correlation between UVSD and PTH levels. According to the literature, HD patients who have high PTH levels also have impaired vascular compliance. PTH may also lead to the impairment of left ventricular diastolic function, and it has been described as an independent predictor of left ventricular hypertrophy (LVH) in HD patients [37,38].

LVH is highly prevalent in the dialysis population. The pathological changes in the heart that lead to LVH present an optimal condition for the onset of arrhythmias and are a significant factor in the occurrence of sudden death in end-stage kidney disease (ESKD) [39]. LVH is an important (and potentially reversible) CV risk factor. Left ventricular mass index (LVMI) is a surrogate of LVH, a predictor of CV morbidity and mortality in hypertensive patients [40]. In our study, high UVSD patients had significantly higher LVMI than low UVSD patients. Pressure overload from hypertension or decreased vascular compliance are major contributors to LVH in ESKD. Preload-related LVH parameters include anemia and volume overload [41]. As BP, hemoglobin and hydration status were not statistically different in our patient groups, we assume these factors were not responsible for the difference in LVH between the groups. On the other hand, according to our results, vascular compliance was better in the high UVSD group, where LMVI was higher.

The size and function of the left atrium can provide valuable insights into the duration and severity of increased left ventricular filling pressures, serving as a potential indicator of mortality in individuals with chronic kidney disease (CKD). An increased left atrial volume index and impaired left atrial strain parameters are both independent predictors of adverse cardiovascular events. Notably, left atrial strain may demonstrate impairment before changes in volume become apparent, making it a promising predictor of both diastolic and systolic function in CKD patients [42,43].

In our study, there was no significant difference between the low and high UVSD group’s left and right atrial sizes and volumes, but we did not measure atrial strain parameters.

Reduced left ventricular ejection fraction (LVEF) defines left ventricular systolic dysfunction [44]. Increased left ventricular end-diastolic diameter (LVEDD) is also a sign of left ventricular dysfunction, and it has been described as a predictor of all-cause mortality in HD patients with coronary artery disease [45,46]. In our study, LVEF was lower, and LVEDD was higher in the high UVSD group than in the low UVSD group, both data indicating LV dysfunction in the high UVSD group. Different studies have emphasized the prognostic role of LV hypertrophy and systolic dysfunction for survival and adverse CV outcomes in CKD patients [47,48,49,50,51].

In our study, more severe cardiac abnormalities were present in high UVSD than in low UVSD patients. Zhang et al. have demonstrated that high visit-to-visit UF volume variability can predict all-cause mortality in HD patients [24]. According to our findings, left ventricular hypertrophy and systolic dysfunction may be responsible for the increased mortality observed in patients with high visit-to-visit UF variability. Surprisingly, in our study, high UVSD patients had somewhat more elastic arteries than the low UVSD patients. We speculate that in this special patient group of HD patients having high visit-to-visit UF variability, there was a dissociation in the development of unfavourable and unfavourable cardiac and vascular abnormalities, with the cardiac lesions occurring before the stiffening of the arteries. The hemodynamic effect of high UF variability might have been more direct on the function and probably later also on the structure of the left ventricle than on the peripheral arteries. High UF variability had detrimental effects on the heart, but it might have postponed the stiffening of the arteries.

## 5. Limitations of the Study

The present study has some important limitations. This was a pilot study that included relatively few patients, which significantly limits the generalizability of the data. We did not measure strain or strain rate by echocardiography.

Our results might be weakened by the cross-sectional method and the single-center nature of the study. In the future, we plan to conduct a follow-up phase of this investigation.

In conclusion, high visit-to-visit UF variability seems to be associated with increased left ventricular hypertrophy and dysfunction and decreased LVEF compared to the condition of low visit-to-visit UV variability despite similar UF volumes temporarily compensated by more elastic arteries. The CV abnormalities observed in high visit-to-visit UF variability may increase the CV risk of these patients.

## Figures and Tables

**Table 1:** Baseline clinical data.

	*HD Patients* *(n = 29)*	*UVSD < 500 mL* *(n = 17)*	*UVSD ≥ 500 mL* *(n = 12)*	*p*
Man/woman (*n*/%)	15/14 (51/48)	7/10 (41/59)	8/4 (67/33)	0.080
Age (year)	67 (57–71)	69.5 (57–76)	64 (57.5–67.75)	0.066
HT (*n*/%)	29 (100)	17 (100)	12 (100)	0.059
DM (*n*/%)	10 (34.4)	4 (23)	6 (50)	0.057
BMI (kg/m^2^)	25.8 (23.5–31.5)	25.1 (22.5–32)	29.2 (25.1–32)	0.250
Hb (g/l)	110.24 ± 11.22	111.80 ± 9.73	107.90 ± 12.27	0.190
Ca (mmol/L)	2.2± 0.15	2.19 ± 0.18	2.21 ± 0.09	0.410
P (mmol/L)	1.7 ± 0.48	1.68 ± 0.48	1.73 ± 0.45	0.630
PTH (pmol/L)	35.97 ± 15.27	32.11 ± 14.93	41.44 ± 13.31	0.340
Glucose (mmol/L)	6.4 ± 1.69	6.73 ± 1.72	5.93 ± 1.46	0.220
Cholesterol (mmol/L)	4.2 (3.7–4.8)	4.2 (3.6–4.5)	4.45 (3.87–4.95)	0.251
Triglyceride (mmol/L)	1.48 (1.18–1.95)	1.63 (1.27–2.03)	1.32 (1.05–1.63)	0.158
HDL cholesterol (mmol/L)	1.1 (0.93–1.46)	1.13 (0.99–1.46)	1.04 (0.92–1.35)	0.237
CRP (mg/L)	4.45 ± 4.29	2.87 ± 2.45	6.70 ± 5.09	0.007
Fe (umol/L)	12.33 ± 3.81	12.51 ± 3.94	12.08 ± 3.42	0.710
Ferritin (mg/L)	311.97 ± 252.93	308.47 ± 192.07	316.91 ± 311.39	0.100
ALP (U/L)	80.1 ± 25.69	80.70 ± 16.51	79.10 ± 33.94	0.591
Creat (umol/L)	719.14 ± 191.97	698.05 ± 169.39	749.00 ± 209.33	0.092
Dialysis vintage (months)	59 ± 34	67.82 ± 37.10	46.50 ± 22.90	0.049
Ultrafiltration volume (ml) single HD	2413.79 ± 766.58	2211.00 ± 729.83	2450.00 ± 781.15	0.092
Ultrafiltration volume/body weight (mL/kg)	7.61 ± 2.14	7.66 ± 2.22	7.55 ± 2.20	0.173
Overhydration assessed by bioimpedance (L)	2.30 ± 1.54	2.21 ± 1.82	2.45 ± 1.16	0.094

UVSD: ultrafiltration volume variability; HT: hypertension; DM: diabetes; BMI: body mass index; Hb: hemoglobin; Ca: calcium; P: phosphate; PTH: parathormone; HDL: high-density lipoprotein; CRP: C- reactive protein; Fe: iron; ALP: alkaline phosphatase; Creat: creatinine.

**Table 2:** Endothelin-1, syndecan-1, systolic and central blood pressure and arterial stiffness parameter (cfPWV, Aix) levels and echocardiographic parameters in the UVSD < 500 mL and UVSD ≥ 500 mL groups.

	*HD Patients* *(n = 29)*	*UVSD < 500 mL* *(n = 17)*	*UVSD ≥ 500 mL* *(n = 12)*	*p*
Brachial systolic/diastolic BP (mmHg)				
pre-HD	141.4/73.3 ± 17.4/17.8	140.7/72.1 ± 19.8/10.2	142.4/75.0 ± 12.2/12.9	0.078
mid-HD	138.4/71.3 ± 19.2/10.1	143.1/71.0 ± 18.7/9.8	131.7/71.9 ± 16.9/10.8	0.056
post-HD	142.9/72.6± 20.2/10.0	147.2/71.7 ± 21.0/10.2	136.9/74.0 ± 16.3/9.2	0.067
Aix (%)				
pre-HD	33.90 ± 9.23	34.11 ± 8.83	33.58 ± 9.38	0.560
mid-HD	33.29 ± 10.91	34.52 ± 11.90	31.36 ± 8.18	0.672
post-HD	31.31 ± 10.02	30.29 ± 9.18	32.75 ± 10.55	0.131
cfPWV (m/s)				
pre-HD	13 (9.37–14.4)	14.1 (10.3–15.4)	10.75 (8.47–12.82) *	0.032
mid-HD	12.25 (9.52–13.92)	12.4 (9.5–17.5)	10.45 (9.5–12.62)	0.076
post-HD	13.77 (10–17.25)	14.3 (10–17.4)	13.9 (10.65–17.75)	0.267
Aorta systolic BP (mmHg)				
pre-HD	130.86 ± 17.37	130.70 ± 19.24	131.08 ± 13.39	0.235
mid-HD	128.41 ± 18.00	133.29 ± 17.53	121.50 ± 15.47	0.041
post-HD	131.45 ± 20.68	134.05 ± 22.55	127.75 ± 15.93	0.076
SDC-1 (ng/mL)				
pre-HD	9.167 ± 2.491	8.64 ± 2.02	9.90 ± 2.78	0.234
mid-HD	9.425 ± 3.008	8.65 ± 2.52	10.43 ± 3.16	0.134
post-HD	9.954 ± 3.180	9.17 ± 2.53	11.05 ± 3.52	0.111
ET-1 (pg/mL)				
pre-HD	9.89 ± 3.48	10.79 ± 2.71	8.61 ± 3.88	0.049
mid-HD	9.92 ± 3.66	10.69 ± 2.65	8.82 ± 4.39	0.098
post-HD	9.61 ± 3.47	10.20 ± 2.63	8.77 ± 4.13	0.087
Echocardiographic parameters				
LVEDD (mm)	52.95 ± 6.53	51.0 ± 5.38	55.7 ± 7.28	0.037
LVESD (mm)	34.1 ± 7.62	32.0 ± 5.61	37.0 ± 9.29	0.091
LVMI (g/m2)	390.12 ± 136.97	350.29 ± 85.92	449.88 ± 180.47	0.038
LVEF (%)	59 (53–66)	60 (55.5–66.5)	53.5 (47–63)	0.036
RAV (mL/m2)	47.63 ± 23.97	43.63 ± 19.82	53.28 ± 27.67	0.054
LAV (mL/m2)	53.90 ± 30.35	51.22 ± 25.12	57.69 ± 35.85	0.131

HD: hemodialysis; UVSD: ultrafiltration volume variability; BP: blood pressure; Aix: augmentation index; cfPWV: carotid femoral pulse wave velocity; SDC-1: syndecan-1; ET-1: endothelin-1; LVEDD: left ventricular end-diastolic diameter; LVESD: left ventricular end-systolic diameter; LVMI: left ventricular mass index; LVEF: left ventricular ejection fraction; RAV: rigth atrial volume; LAV: left atrial volume. * pre-HD cfPWV vs. post-HD cfPWV, *p* = 0.029.

## Data Availability

The data in this article cannot be publicly shared due to Hungarian regulations and the need to protect the privacy of study participants. However, the data can be made available upon reasonable request to the corresponding author, provided it is accepted by the Regional Committee for Medical and Health Research Ethics and local Data Protection Officials.
